# Origins of obesity in the womb: Fetal adiposity and its determinants

**DOI:** 10.1111/jog.16114

**Published:** 2024-10-09

**Authors:** Satoru Ikenoue, Junko Tamai, Keisuke Akita, Toshimitsu Otani, Yoshifumi Kasuga, Mamoru Tanaka

**Affiliations:** ^1^ Department of Obstetrics and Gynecology Keio University School of Medicine Tokyo Japan

**Keywords:** DOHaD, fetal adiposity, fetal body composition, fetal ultrasonography, fractional limb volume, liver blood flow

## Abstract

Birth weight is an important predictor of perinatal complications and long‐term health outcomes of offspring. Fetal programming influenced by maternal obesity, overnutrition, and hyperglycemia has been proposed as the fuel overload hypothesis. Recent investigations related with fetal body composition have revealed that neonatal adiposity can be predicted by fetal fat mass, and that maternal insulin resistance and serum leptin level are indicators of fetal adiposity. Based on the current evidence, the origins of obesity can partly be traced back into the fetal life. Further clarification of the determinants of fetal fat mass may lead to the clinical interventions and treatment strategies for fetal growth and development. This effort potentially leads to the elucidation of pathological conditions related with long‐term health outcomes and the primary prevention of childhood obesity and early onset metabolic syndrome.

## INTRODUCTION

It is well established that birth weight is an important predictor of perinatal complications and long‐term health outcomes of the offsprings (Developmental Origins of Health and Disease). In the 1980s, Barker et al. epidemiologically revealed that low birthweight infants have a higher risk of cardiovascular disease and metabolic syndrome in the adulthood,^1^ which is called “Thrifty hypothesis” that maternal undernutrition affects the intrauterine fetal programming. Boney et al. also reported that heavy‐for‐date infants born from mothers with gestational diabetes mellitus (GDM) have an increased risk of obesity, hypertension, hyperlipidemia, and glucose intolerance in early childhood.^2^ Additionally, the hyperglycemia and adverse pregnancy outcome (HAPO) study conducted with 23 316 mothers demonstrated a linear positive correlation between maternal blood glucose level and neonatal body fat percentage.^3^, ^4^ HAPO follow up study with 4832 followed‐up children indicated that in the GDM group as compared with normal glucose tolerance (NGT) group, body mass index, abdominal circumference, subcutaneous fat mass, and body fat percentage were significantly higher at age 11 years.^5^ Based on these evidence, fetal programming influenced by maternal obesity, overnutrition, and hyperglycemia has been proposed as the “fuel overload hypothesis.” That is, excessive nutrient supply from the mother to the fetus suppresses fatty acid oxidation and glycogenesis in the fetus, which leads to increased fetal adiposity and subsequent childhood obesity and early onset metabolic syndrome.^6^

### Fat deposition in the human fetus

Fat mass is one of the important components of body composition. Particularly, human neonates have higher body fat percentage than other mammals. Approximately 46% of the variation in individual differences of birth weight are explained by fat mass.^7^ High percentage body fat of infant is considered as a result of the evolutionary process of the human being. Fat mass was crucial for human infants for (1) maintaining body temperature after birth with little body hair, (2) providing an energy source for starvation, and (3) secondary energy source for the brain, which is significantly larger in the human infants than other mammals.^8^

Fetal fat accrual begins around 16 weeks and rapidly increases after 30 weeks, with more than 80% of fat mass is accumulated in the third trimester.^9^ Neonatal body fat percentage significantly correlates with childhood adiposity.^10^ Hence, neonatal body fat percentage is a potential predictor of childhood obesity. Dual energy X‐ray absorptiometry and air displacement plethysmography are the golden standards for measuring neonatal body fat percentage.^11^ However, there have been few reports evaluating fetal adiposity.

The conventional golden standard for evaluating fetal growth has been the estimated fetal weight calculated by ultrasonography, which has a measurement error of ±10%. This is partly because the estimated weight is calculated mainly based on the bone components (e.g., biparietal diameter and femur length), and soft tissue volume including fat mass and lean mass have not been considered.

### Fetal fractional limb volume

Since the measurement error of estimated fetal weight tends to be particularly large in infants with large‐for‐gestational age (with increased soft tissue volume), novel ultrasound parameters that reflect fetal soft tissue volume have been warranted. Lee et al. introduced fetal fractional limb volume measures as a new indicator of fetal growth including fat mass and lean mass. Fractional limb volume is assessed as cylindrical limb volumes based on 50% of the fetal total diaphysis length using 3D ultrasonography. Fractional arm volume and thigh volume increase linearly during the second trimester while their growth are accelerated in the third trimester of gestation.^12^, ^13^, ^14^ Additionally, fractional limb volume has been reported to be useful in predicting birth weight and neonatal body fat percentage.^15^, ^16^

It is well known that infants born to mothers with impaired glucose tolerance have an increased risk of shoulder dystocia as a result of increased fat mass in the fetal upper arm and enlarged shoulder circumference.^17^ In a retrospective study, fractional arm volume has been reported to be significantly larger in the GDM group after 32 weeks' gestation while no significant difference was observed in fractional thigh volume across gestation.^18^ Moreover, there was no difference in estimated fetal weight or birth weight between GDM and NGT. It has also been reported that fat mass was significantly increased in GDM infants even though maternal glycemic control was good and the infant birth weight was appropriate for gestational age.^19^ Similarly, fat mass in the upper‐arm were significantly increased in the GDM group compared with the NGT group in the third trimester although there was no difference in skeletal muscle mass.^20^, ^21^ Based on these findings, fetal upper arm volume could be an early indicator of altered fetal growth in GDM women.

### Fetal fat mass measures and the concept of estimated fetal adiposity

Fetal fractional limb volume is a novel parameter for evaluating fetal soft tissue volume, however, it includes not only fat mass but also lean mass and bone. Measures of fetal subcutaneous fat mass have been reported in fetal limb, abdomen, cheeks, buttocks, and subscapular areas. Particularly, upper arm, thigh and abdomen are sites where fetal fat mass can be measured reproducibly.^22^

Previous reports have primarily used single‐site measurements of fetal subcutaneous fat in the extremities and abdomen.^12^, ^13^ To derive the index of total fetal adiposity, a composite measure of fetal adiposity using arm percent fat area, thigh percent fat area and anterior abdominal wall thickness has recently been introduced. Because these parameters have different units of measurement, each measurement units was converted to standardized (*z*) scores, then the mean of the three *z* scores was computed as a composite estimate of fetal adiposity (EFA). Using this novel parameter, we have reported that EFA at 30 weeks gestation significantly correlates with newborn adiposity. Particularly, fat mass in the upper arm was most useful in predicting increased neonatal body fat percentage.^23^

### Determinants of fetal adiposity

#### Maternal insulin resistance and fetal fat mass

To provide clinical intervention for fetal growth and development using EFA, it is necessary to clarify its determinants. As mentioned above, HAPO study has shown that maternal blood glucose levels are associated with neonatal body fat percentage. In a prospective study of 137 singleton pregnant women, maternal insulin resistance at 20 weeks was already associated with fetal fat deposition (EFA) at 20 weeks or later even after controlling for the confounding factors.^24^ Hence, maternal insulin resistance in the second trimester of pregnancy could be an indicator of fetal adiposity. There are several possible mechanisms by which maternal insulin resistance increases fetal fat mass. First, maternal blood glucose is diffused and transported to the fetal circulation via placental glucose transporters (GLUTs). Fetal hyperglycemia causes pancreatic beta cell hyperplasia and hypertrophy, which in turn causes fetal hyperinsulinemia.^25^ Fetal hyperinsulinemia suppresses triglyceride degradation and promotes triglyceride synthesis in the liver, resulting in an increased fetal fat deposition. Second, in pregnant women with impaired glucose tolerance, placental lipoprotein lipase is activated and de‐esterification of triglyceride is accelerated. Free fatty acids from the degradation products are transported to the fetal side by placental fatty acid binding proteins and fatty acid transporters, resulting in increased fatty acid supply to the fetal circulation.^26^ These mechanisms are considered to increase fetal fat mass in association with increased maternal insulin resistance.

#### Serum leptin level and fetal fat mass

Leptin is an adipocytokine derived from adipocytes and involved in lipid metabolism such as fatty acid oxidation. Leptin is also known to be closely interactive with insulin sharing signaling pathways (e.g., MAPK, PI3K, and JAK2/STAT‐3).^27^, ^28^, ^29^ It has also been reported that elevated maternal serum leptin increases cord blood C‐peptide, an indicator of fetal insulin resistance.^30^ In fact, cord blood leptin level has been reported to relate to fetal abdominal fat at 34 weeks.^30^

#### Fetal liver blood perfusion as a possible determinant of fetal adiposity

Fetal liver is the primary organ that controls nutrients synthesis and degradation of major nutritional substrates (carbohydrates, lipids, and proteins). The blood flow from the placenta to the fetus is shunted through the ductus venosus to the fetal heart and brain, and the rest of the blood flow perfuses the fetal liver. Fetal liver is an important organ for lipid metabolism, where triglycerides are synthesized by de novo lipogenesis and stored as adipose tissue for energy storage.^31^ Especially, the acetyl CoA carboxylase, fatty acid synthase and glucose‐6‐phosphate dehydrogenase are activated in the fetal period.^32^, ^33^

In animal experiments with sheep, increased fetal hepatic blood flow accelerates hepatocytes secretion of insulin‐like growth factor 1/2 (IGF‐1/2) and hepatocyte growth factor, which are related to glucose and lipid metabolism and fetal development.^34^, ^35^, ^36^ Hepatic blood flow can be calculated in human fetus using ultrasonography as the difference between umbilical venous blood flow and ductal venous blood flow.^37^, ^38^, ^39^ Fetal liver blood flow at 30 weeks has been associated with neonatal adiposity (percentage body fat), but not with lean mass.^40^ Godfrey et al. also reported that fetal hepatic blood flow at 36 weeks correlated with body fat percentage at birth and at 4 years old.^37^ These findings suggest that increased fetal liver blood flow promotes triglyceride synthesis and increases fetal fat deposition.

#### Determinants of fetal liver blood flow

Maternal or placental factors that potentially influence fetal liver blood flow and fetal fat mass include maternal pregravid body mass index, gestational weight gain, serum markers related to glucose and lipid metabolism, placental transporters of carbohydrate and lipids, and placenta‐derived hormones. Corticotrophin releasing hormone (CRH) is a peptide hormone that plays a central role in the hypothalamus‐pituitary‐adrenal axis and is secreted in response to physiological stress.^41^ CRH is also secreted by the placenta during pregnancy and is detected in maternal circulation. The secretion of CRH from the placenta (placental CRH) increases approximately 100‐fold in the third trimester of pregnancy.^42^ CRH is known to relax vascular smooth muscle in the skin, lung, and kidney, and it also has vasodilatory effects in the human fetal‐placental circulation.^43^ Placental CRH has also been associated with fetal growth and infant obesity.^44^, ^45^ In our prospective study, placental CRH at 30 weeks showed a significant positive correlation with fetal liver blood flow volume.^46^ Placental CRH has been reported to increase the expression of glucose transporters in placental trophoblasts in a dose‐dependent manner.^47^ Although it is still unclear whether placental CRH has direct effect on fetal fat deposition, higher placental CRH level could increase fetal liver blood flow and glucose supply from the mother to the fetus, which subsequently increase fetal adiposity.

### Future implications

Estimated fetal weight, which has been used as the main indicator of fetal growth for more than 50 years, is difficult to distinguish constitutionally large fetus with uncomplicated fetal growth and perinatal outcomes from pathologically large fetuses with higher risk of shoulder dystocia due to increased adiposity. Fetal soft tissue volume, especially fat mass, is now increasingly focused as a novel ultrasonographic parameter of fetal growth and body composition. Further investigations are warranted to clarify the determinant of fetal adiposity including the maternal background (age, parity, body mass index,^48^ gestational weight gain^49^), serum biomarkers (glucose and lipid metabolism), placental factors (carbohydrate and lipid transporters), and stress markers (cortisol, CRH). These efforts may enable the establishment of clinical interventions and treatment strategies to improve fetal development and long‐term health outcomes of offsprings.

## CONCLUSION

Based on the recent investigations of fetal body composition, it has been revealed that fetal fat mass can predict neonatal adiposity, and that maternal insulin resistance and serum leptin level could be indicators of fetal adiposity. Additionally, fetal hepatic blood flow and placenta‐derived CRH potentially are important parameters for fetal fat accumulation. Since body fat percentage in the neonatal period is associated with childhood obesity, the origins of obesity can be partly traced back into the fetal life. Further clarification of the determinants of fetal fat mass will elucidate the mechanism of fetal fat accumulation, which may lead to the introduction of clinical interventions and treatment strategies for fetal growth and development. Evaluating fetal growth and body composition using novel fetal ultrasound parameters (e.g., fetal fat mass), with long‐term follow‐up of the infants, potentially lead to the elucidation of pathological conditions related with long‐term health outcomes after birth and the primary prevention of childhood obesity and early onset metabolic syndrome.

## CONFLICT OF INTEREST STATEMENT

The authors report there are no competing interests to declare. Dr. Yoshifumi Kasuga is an Editorial Board member of JOG Journal and a co‐author of this article. To minimize bias, Dr. Kasuga was excluded from all editorial decision‐making related to the acceptance of this article for publication.

## Data Availability

Data sharing is not applicable to this article as no new data were created or analyzed in this study.
